# Elementary school staff perspectives on the implementation of physical activity approaches in practice: an exploratory sequential mixed methods study

**DOI:** 10.3389/fpubh.2023.1193442

**Published:** 2023-08-24

**Authors:** Timothy J. Walker, Christopher D. Pfledderer, Derek W. Craig, Michael C. Robertson, Natalia I. Heredia, John B. Bartholomew

**Affiliations:** ^1^The University of Texas Health Science Center at Houston School of Public Health, Houston, TX, United States; ^2^The University of Texas Health Science Center at Houston School of Public Health, Austin Regional Campus, Austin, TX, United States; ^3^The University of Texas Medical Branch, Galveston, TX, United States; ^4^Department of Kinesiology and Health Education, The University of Texas at Austin, Austin, TX, United States

**Keywords:** physical activity, implementation, whole-of-school, whole-school, school

## Abstract

**Introduction:**

A whole-of-school approach is best to promote physical activity before, during, and after school. However, multicomponent programming is often complex and difficult to deliver in school settings. There is a need to better understand how components of a whole-of-school approach are implemented in practice. The objectives of this mixed methods study were to: (1) qualitatively explore physical activity approaches and their implementation in elementary schools, (2) quantitatively assess implementation levels, and (3) examine associations between school-level physical activity promotion and academic ratings.

**Methods:**

We used an exploratory sequential mixed methods design. We conducted semi-structured qualitative interviews with elementary school staff from a Texas school district and used a directed content analysis to explore physical activity approaches and their implementation. Using qualitative findings, we designed a survey to quantitatively examine the implementation of physical activity approaches, which we distributed to elementary staff district wide. We used Pearson correlation coefficients to examine the association between the amount of physical activity opportunities present in individual schools and school-level academic ratings.

**Results:**

We completed 15 interviews (7 principals/assistant principals, 4 physical educators, and 4 classroom teachers). Elementary school teachers and staff indicated PE and recess implementation was driven from the top-down by state and district policies, while implementation of classroom-based approaches, before and after school programming, and active transport were largely driven from the bottom-up by teachers and school leaders. Teachers and staff also discussed implementation challenges across approaches. Survey respondents (*n* = 247 from 22 schools) indicated 54.6% of schools were implementing ≥135 min/week of physical education and 72.7% were implementing 30 min/day of recess. Classroom-based approaches were less common. Twenty-four percent of schools reported accessible before school programs, 72.7% reported accessible after school programs, and 27% promoted active transport. There was a direct association between the number of physical activity opportunities provided and school-level academic ratings *r*(22) = 0.53, *p* = 0.01.

**Conclusion:**

Schools provided physical activity opportunities consistent with a whole-of-school approach, although there was variability between schools and implementation challenges were present. Leveraging existing school assets while providing school-specific implementation strategies may be most beneficial for supporting successful physical activity promotion in elementary schools.

## Introduction

Schools play vital role in supporting student’s physical activity (PA). On a given week, about 1 billion children across the world attend school, where they spend a majority of their daytime hours (1). Effective school-based PA programming has the potential to improve student’s health, well-being, and academic performance (2–4). Due to reductions in physical education (PE) and recess over time, The Institute of Medicine and other authorities recommend schools use a whole-of-school approach for PA that includes promoting active travel to and from school, before/after school programs, recess and lunchtime breaks, physical education (PE), and PA during classroom instruction time (1, 5). A whole-of-school approach involves school leaders, teachers, and other staff to coordinate PA opportunities throughout the day while also maintaining core academic responsibilities.

The Comprehensive School Physical Activity Program (CSPAP) conceptual framework provides additional guidance about a whole-of-school approach by using a social ecological perspective to highlight different sources of influence (6). Numerous school-based interventions have been informed by the CSPAP framework or address its components. Despite the promise of CSPAPs, school-based PA interventions have had limited success, which has been attributed to ineffective intervention components and/or poor implementation (7–9). Many school-based interventions are complex leading to significant implementation challenges (7, 8). Successful implementation is often dependent on a busy school staff dealing with competing academic priorities (10). The challenge of implementation can be made worse when there is a failure to consult with end users during the design process (11). Far too often, interventions are imposed on teachers and staff who are expected to prioritize the intervention over other obligations. The lack of input from teachers and staff can lead to interventions that fail to address school needs or lack long-term sustainability.

To improve PA promotion in schools, there is a need to better understand the approaches schools use in practice, how they are implemented, and how they relate to a school’s academic performance. Despite existing research supporting the link between student PA and academic outcomes, less is known about how PA opportunities implemented by schools relate to their overall academic performance. Gaining a better understanding of current practice can help identify research needs and inform how the existing school-based research can guide practice-based efforts. Therefore, the objectives of this study are to: (1) explore PA approaches used in elementary schools and how they are implemented; (2) assess implementation trends of PA approaches across a large, highly diverse district; and (3) examine the association between school-level PA promotion and school-level academic ratings.

## Methods

### Study design

**Figure 1:**
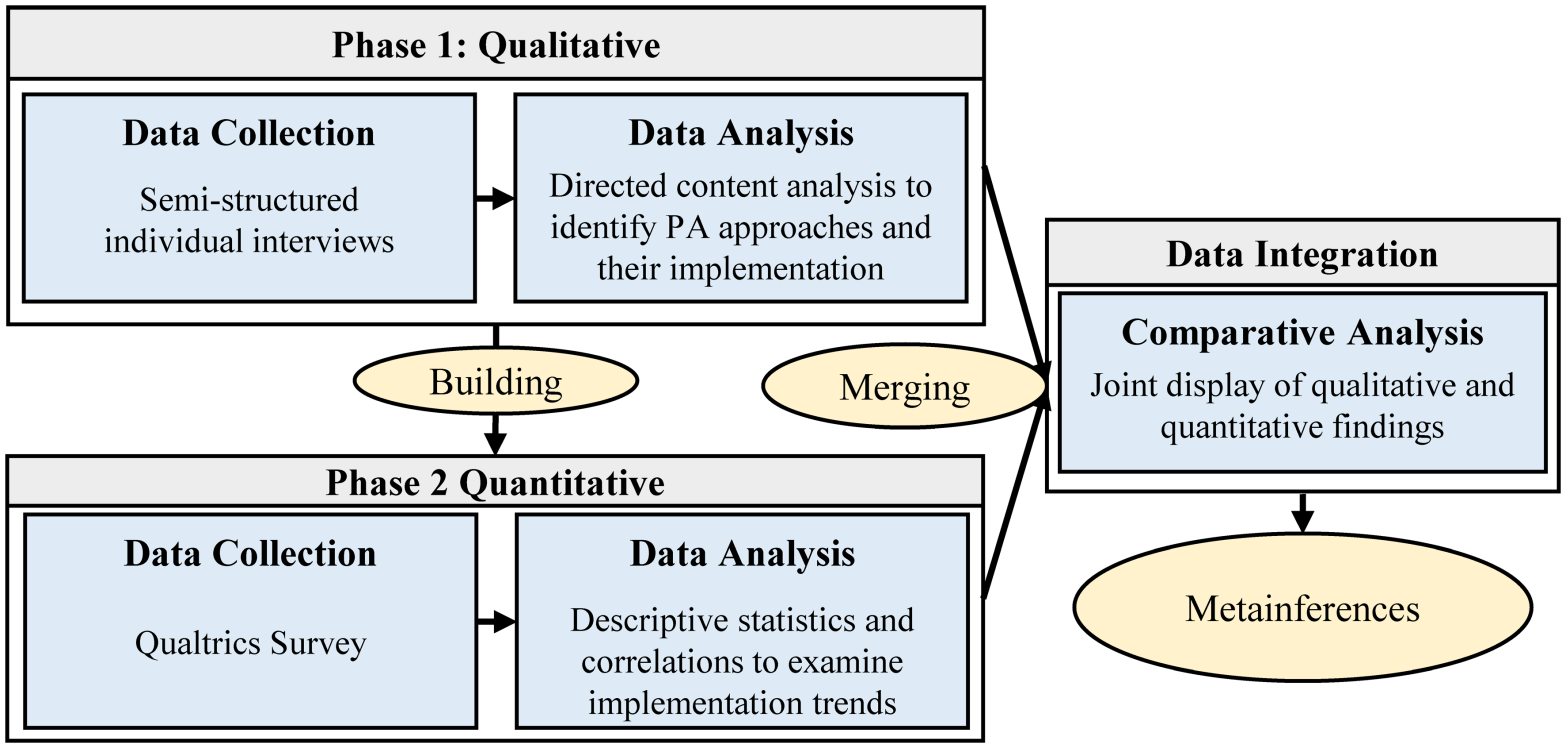
Exploratory sequential mixed methods design.

We used an exploratory sequential mixed methods design with a qualitative phase (Spring 2018) followed by a quantitative phase (Summer/Fall 2019) (Figure 1). During the qualitative phase, we explored PA opportunities provided by elementary schools and how they were implemented. We used qualitative findings to develop a survey and distribute to elementary school staff throughout a large, diverse district. We designed the survey to examine PA implementation trends across the elementary schools in the district. We merged qualitative and quantitative findings using a joint display to enhance our understanding of PA implementation. We also integrated school-level academic data from the Texas Education Agency (TEA) to further investigate school-level PA implementation and academic ratings. The Committee for the Protection of Human Subjects at The University of Texas Health Science Center Houston approved this study.

### Qualitative phase

#### Participant recruitment for interviews

We used a purposeful sampling approach to recruit interview participants. Participants were eligible for the study if they worked at an elementary school and could speak to the PA opportunities provided at their school. We asked district-level wellness staff to provide contact information for elementary school staff who knew the PA opportunities at their school. Research staff then sent emails to potential participants to arrange interviews. We recruited additional participants by asking interviewees to provide contact information for their colleagues. As study enrollment increased, we focused recruitment efforts to balance participants across four job types: principals, assistant principals, PE teachers, and classroom/support teachers (e.g., multiclass room leaders). We determined the final sample size for interviews based on pragmatic considerations of the study goals, data quality, and achieving data saturation (i.e., when new participants produced minimal new information) (12).

#### Qualitative data collection

We completed semi-structured interviews during Spring 2018, using an interview guide that included questions about PA approaches used in schools and how they were implemented (see Additional file 1). At the end of each interview, we collected demographic information (job type, gender, age, years of experience). The lead author completed all interviews in-person and audio recorded them. A professional transcription company transcribed the audio files verbatim. Each interview participant received a $30 gift card.

#### Qualitative data analysis

We conducted a directed content analysis to explore PA approaches used by schools (13). We used the whole-of-school approach to inform coding and analysis by applying deductive codes for its components, and inductive codes for approaches not specified by a whole-of-school approach (5). Three members of the research team coded transcripts using Dedoose (14). They first coded three transcripts independently, and then met to discuss codes and reconcile discrepancies. After establishing consensus, the lead author coded the remaining 12 transcripts and the other two researchers each coded six. The team met throughout the coding process to discuss discrepancies and new codes. We further analyzed codes by reviewing and summarizing interview excerpts for each coded topic area.

### Quantitative phase

#### Participant recruitment for surveys

Per district policy, we contacted elementary school principals about the survey and provided an opportunity to decline participation for their school. After determining the final list of schools, we obtained email addresses from each participating school’s websites. We distributed the electronic (Qualtrics) survey via email to elementary school staff in Summer/Fall 2019. The email included a letter of information about the study and participants provided their consent by selecting the survey link. Elementary school staff were eligible to complete the survey if they worked at a participating elementary school in the 2018–2019 school year, worked with kindergarten-5th grade students, and had a valid district email address. The first 500 respondents were eligible to receive a $30 gift card.

#### Quantitative data collection

We used qualitative findings and feedback from district-level wellness partners to inform survey questions for the quantitative phase. Specifically, we included survey questions about the school’s use of PA opportunities consistent with a whole-of-school approach: PE, recess, classroom-based PA approaches, before school programs, after school programs, and active travel (Table 1). The participating school district used the term health fitness to refer to PE, which is reflected in the survey questions. We also included questions for teacher’s individual respondent characteristics (i.e., job title, years in current position, years working in education, gender, and age).

**Table 1:** Quantitative survey questions for whole-of-school components.

Survey questions	Response options	Scoring
**Physical education**		
Q1 In a typical week, how many days do students at your school attend health fitness^a^ class? (If your school uses an alternating schedule, you may use decimals. For example, if the health fitness schedule alternates between 2 and 3 times every week, you may enter 2.5)	Number of days/week	Mode (min/week) Low: <135 min/week Med: ≥135 min over 2 weeks^b^ High: ≥135 min/week
Q2. How many minutes is a typical health fitness class?	Number of minutes	
**Recess**		
Q3. In a typical week, how many days do students at your school have a scheduled recess period?	Number of days/week	Mode (min/week) Low: <100 min/week Med: 100–149 min/week High: ≥150 min/week
Q4. How many times per day do your students go out for recess?	Number of times/day	
Q5. How many minutes are the scheduled recess periods?	Number of minutes	
**Classroom-based approaches**		
Q6. To what extent were the following programs or approaches for physical activity used in the 2018–2019 school year?	1) Not used by any 2) Used by some 3) Used by about half 4) Used by most 5) Used by all 6) Do not know/not sure	Mean (1–5 Likert-type scale) Low: <3 Med: 3–3.9 High: ≥4
(a) Classroom-based physical activity approaches (active learning lessons, brain breaks, or GoNoodle done in the classroom)		
(b) Motor labs or action-based learning labs (a designated space with equipment to do active learning lessons)		
**Afterschool, before school, active transport**		
Q7. During the 2018–19 school year…	1) Strongly agree 2) Somewhat agree 3) Neither agree nor disagree 4) Somewhat disagree 5) Strongly disagree	Mean (1–5 Likert scale) Low: <3 Med: 3–3.9 High: ≥4
(a) We had after-school programs that were accessible to all students		
(b) We had before-school programs that were accessible to all students (e.g., a morning run club)		
(c) We encouraged students who lived nearby to walk or bike to school		
(d) We had an active commuting to school program that was accessible to all students (e.g., organized walking/biking to school, safe routes to school, or walking school bus^c^)		

a Participating district referred to physical education class as health fitness class.

b Schools used an alternating PE schedule that was <135 min 1 week, and >135 min another week.

c Active transport questions were averaged together for each individual respondent and then averaged within their respective school.

#### Quantitative data processing

We cleaned and analyzed survey data using Stata 15.0. We examined descriptive statistics for survey participants using the individual characteristics questions. We used publicly available data from TEA to provide school-level characteristics (i.e., percentage of economically disadvantaged students). We screened for and removed ineligible respondents (e.g., pre-k teachers) and unrealistic values from the data (e.g., reported PE of 1 min/class).

##### Scores for PA opportunities

We created and reported school-level values for each respective PA opportunity within a whole-of-school approach. We used the most common answers (mode) from respondents to represent a school’s days and minutes per week of PE and recess. The mode provided a meaningful value for weekly days and minutes for each school, and provided consistent results to other measures of central tendency (means and medians). We then categorized schools based on their fulfillment of Texas’s PA policy, which requires a minimum of 135 min of structured physical activity per week (15). While the intention of the policy is for this to be PE, schools are able to substitute other forms of supervised activity (e.g., walking on the school track). The PE categories were: (1) <135 min/week; (2) ≥135 min but averaged over 2 weeks due to an alternating schedule; and (3) ≥135 min/week. For recess, we categorized schools based on weekly minutes of recess: (1) <100 min/week; (2) 100–149 min/week; and (3) ≥150 min/week.

We generated similar, school-level scores for: classroom-based approaches, before and after school programs, and active transport (i.e., students walking or biking to school). We calculated the mean of responses within each school because the corresponding questions for these components used a 5-point Likert response scale (16). We also categorized schools into low (<3), medium (3–3.9) and high (≥4) categories for each respective variable.

##### Total PA score

We created a PA index score by adding values across the opportunities promoted in a whole-of-school approach. Schools received a 0 for low, 1 for medium, and 2 for high values across each respective type of PA opportunity provided.

##### Academic rating

We obtained each school’s academic accountability rating from the TEA website (17). The overall accountability rating is scored on a 100-point scale, translated to an A–F rating (<60 = F; 60 to <70 = D; 70 to <80 = C, 80 to <90 = B; ≥90 = A), and based on three domains: student achievement, school progress, and closing achievement gaps (17).

#### Statistical analysis

We examined values across each school-level variable, and used a scatter plot and Pearson correlation coefficient to examine the association between the school PA index score and the accountability rating.

### Mixed methods

We used mixed methods integration strategies during data collection such as building (developing survey contents from qualitative findings) and exploring (using a qualitative approach to understand PA opportunities implemented in schools prior to conducting a quantitative study to confirm findings) (18). We also used integration procedures for data analysis by merging qualitative and quantitative data using a joint display and to enhance our understanding of PA implementation across schools in our partner district (18).

## Results

### Qualitative results

We completed 15 interviews during the qualitative phase (four principals, three assistant principals, four PE teachers, and four teachers). Interview participants were from 10 different elementary schools across the district. Almost all participants were female (93%) and had an average of 8.5 years of experience in their current position.

#### Types of PA approaches

Schools were using multiple PA approaches throughout the school day. Almost all approaches aligned with components of a whole-of-school approach (Table 2). Schools also provided PA approaches through “specials” rotations (i.e., noncore courses such as music, art, or library attended in rotations), and through single day events (e.g., a field day).

**Table 2:** Physical activity approaches used by schools.

WOS^a^ approach	Description	Examples^b^
Physical education	Structured classes led by a certified teacher to develop students’ physical competence	District supported curriculums (SPARK^c^, CATCH^d^) Open-source curriculums Lessons guided by TEKS^e^
Recess	Regularly scheduled periods within the school day for supervised physical activity and play	Unstructured play time Students run laps, then have free play time Teacher-organized activities (e.g., soccer club at recess) Structured recess programs (e.g., Playworks)
Classroom-based	Opportunities provided in the classroom as a break from, or part of instruction time	Flexible seating (wobble chairs, stability balls, pedal desks) Brain breaks (movement breaks mostly facilitated by GoNoodle, Music, etc.) Physically active learning (marching while singing phonics songs) Motorlabs (designated spaces with movement station rotations for physically active learning)
After school programs	Programs provided afterschool that promote physical activity	Onsite programs organized by school (e.g., tennis, basketball, running clubs, soccer) Onsite community partnership programs (YMCA, BGCGH^f^) Offsite community partnerships (e.g., students are bussed to a local church program)
Before school programs	Programs provided before school that promote physical activity	Open gym time or outside activities Morning walking/running clubs Motorlabs made available before the school day
Active transport	Promotion of walking, biking, or other forms of physically active transport to school	Walk to school day
Events	One-time events that promote or engage students in physical activity	Health fairs, Field days, field trips, fun runs Invited guest events (e.g., professional soccer engaging students in activity)
Specials rotations	Noncore classes that intentionally engage students in physical activity	Music class designed to engage students in movement (e.g., dancing or other forms of movement)

a WOS stands for whole-of-school.

b Examples come from qualitative interviews with participants.

c SPARK stands for sports, play, and active recreation for kids.

d CATCH stands for coordinated approach to child health.

e TEKS stands for Texas essential knowledge and skills.

f BGCGH stands for boys and girls club of greater Houston.

##### Physical education

Participants indicated the allocated PE time was primarily driven by state policy. School leaders reported how they set the PE time and would submit their schedule to district staff for review, who would then submit it to the state. Participants indicated some schools consistently met or exceeded the policy, while some fell below. Other schools used a rotating schedule, three sessions 1 week, which met state policy, followed by a week with two sessions, which fell short of state policy. There were mixed reports about how well the PE policy was enforced at the state, district, and school levels. For example, when discussing the role schools play in supporting PA opportunities, a PE teacher (Participant 2) explained: “It’s to continue with reinforcing the 135 min (of PE), a lot of schools do not. I’m glad we do.”

School leaders explained how time, limited resources, and competing priorities impacted PE scheduling. One principal (Participant 12) explained: “If I had all the personnel I needed, we would have it (PE) every day…we are required to have so many minutes a week, and we meet those guidelines for the state. But if I could extend the day another 30 min or an hour, then we could build that into every day….” Another principal (Participant 10) explained how they were reducing PE time to create more space for electives: “Next year, we are actually changing our schedule. Our kids will not get 135 min of just health fitness. They will get about 125, and the other ten or more if we chose will be through things—because they do GoNoodle, they go to motorlabs, they go to music, they go to recess for 30 min, and so cutting off time at specials is also providing us an opportunity once a week for 2nd–5th grade to participate in an elective next year, and so we hope to offer things like yoga and dance and theater and chess, robotics…..”

##### Recess

Recess time was driven by district rather than state policy. Even though all schools were from the same district, some participants reported recess policy to be 20 min/day whereas others reported 30 min/day. School leaders set the recess time and schedule. Teachers implemented this schedule, although the policy was not always enforced as a PE teacher explained (Participant 1): “There’s a district policy requiring recess and the amount of minutes…but following up on that (recess policy) and not letting teachers opt out.” A principal (Participant 12) further explained the challenges of enforcing the recess policy among teachers: “I may have a parent call and say, ‘My child’s not going to recess because they needed to finish their work.’ Well, you know, we have over fifty teachers here in this building, and I cannot be in every classroom with them all day.”

Students sometimes lost recess time because of poor behavior, to make up work, or for whole-class restroom breaks. For example, a classroom teacher (Participant 9) explained how they were encouraged to do whole-class restroom breaks during recess rather than class time: “Teachers were told that if you need to take your class to the restroom that that has to be a part of that 30 min (recess time).” The teacher (Participant 9) further explained how it was important to have buy-in from the entire staff for recess: “…they (the whole staff) all need to have buy-in into why recess is so important, and not be told that ‘you have to take the kids out for recess because that’s what’s good for them.’ Because even with our kids, if we are telling them to do something, it does not mean anything, but showing them why they have to do it, that hits them more, so they are more likely to do it.”

##### Classroom-based approaches

Schools were using different types of classroom-based approaches including flexible seating, brain breaks, and physically active learning (Table 2). These approaches were largely up to the teachers to implement as there were no reported district or school policies. Brain breaks are short, PA sessions to provide a break from traditional didactic instruction. For example, a teacher may play a brief dance video to allow their students to engage in PA between academic lessons. Some schools struggled to get a majority of teachers using brain breaks as one PE teacher (Participant 4) explained: “They (teachers) need to be bought in, but they just—they have not. And there’s no consequence or something coming down from the administrator that’s saying, ‘When I walk in your classroom, I want to see these things.’” At other schools, leadership support improved the use of brain breaks as an assistant principal (Participant 3) described: “Instead of having them (students) be off task and get in trouble because they are moving—let us put that as part of their day, so there’s a reason and purpose for movement, and then they can refocus. When we framed it like that, the teachers were like, ‘Okay, we’ll try this.’ And the more they tried it, the more they saw, ‘Oh, this really does work.’ And now, it’s part of the culture of the building.” Participants described numerous resources to aid the use of brain breaks (Table 2), how they were incorporated into class transitions, and how they were more commonly used among kindergarten-2nd grade teachers compared to 3rd–5th grade teachers.

Physically active learning occurs when integrating movement into academic lessons (e.g., having students act out the definition of a word as part of the learning lesson). The use of physically active learning was at the teacher’s discretion, and these lessons required more planning compared to brain breaks. One teacher (Participant 6) explained how she shifted a lesson and the impact it had on students: “…I had to switch gears and thinking, okay, how can I incorporate movement, and we have done the same phonics song since the first day of school. All I did was add a march to it, and it was reborn. They were so excited. ‘Can we please march? Can we please march?’…So, all I did was simply add that march to it, and they are revved up ready to go.”

Motorlabs are designated spaces with ready-to-use equipment (usually set up in stations) to facilitate physically active learning lessons. For example, a station may have a rope latter for students to jump through while reading different site words. Motorlabs required resources, teacher training, and time to bring students to the lab. One principal (Participant 10) explained the progression to incorporate academic content into the motorlab: “The first couple of weeks they take them in to teach them how to rotate and how to perform the activity at that station. Then the teachers start to incorporate sight-words, letter sounds, spelling patterns, sentence patterns, math facts, and so in the room you’ll see things switched around by each station that they want the kids to practice.”

The decision to set up a motorlab often went through school leaders, and once set up, schools used different approaches for promotion. For example, a teacher (Participant 11) explained how they scheduled time in the motorlab: “motor lab is an open schedule and you can put yourself in 20, 30 min slots. You may come as many times as you want during the week as long as somebody is not in that slot.” Given that many teachers did not use the lab on their own, some schools tried to include the motorlab in the “specials” rotation and have a PE teacher oversee it. Including the motorlab as part of the “specials” rotation had tradeoffs as a PE teacher (Participant 4) explained: “I think it just waters down both programs (motorlabs and PE). I do not see the kids academically, so I can guess where they are, where they should be, but I’m not focused specifically on a student’s need because I’m not their classroom teacher. And then the health fitness is watered down because you have one instructor planning for 760 kids and we do not see them as often.”

##### Active travel, before school, and after school programs

Participants reported that schools provided active travel events, and before and after school programs (Table 2). Some schools promoted active travel by having a walk-to-school day where kids would walk to school and be served treats. Participants also highlighted how before school programs required staff to organize them, as one assistant principal (Participant 15) explained: “In the mornings, our dean of students started a walking program for the kids. It’s between 7:00 and 7:15 in the morning. It’s only 15 min, but I feel like it’s pretty neat.” Some schools did not have before school programs or were in the process of initiating them as a principal (Participant 13) explained: “In the mornings, we are starting to give our kids options. We’re calling it Morning Menu. Right now, our kids show up at seven o’clock when we open the building. And so, they go to the cafeteria or the gym. And basically, what they do is they eat in the cafeteria, and then they go and they sit in the line and wait for their teachers to show up at 7:25.” The principal (Participant 13) further explained how they are starting the Morning Menu: “So, right now we are also working on what that process will look like because you are going to have to have people—the logistics. You have to have people supervising the kids. And then, you also have to teach the kids how to—you know—evaluate, set their own goals.”

There were also after school programs that school leaders and/or staff arranged (Table 2). The after school programs were offered onsite and offsite through community partnerships. Some schools did not have formal after school programs and relied on teacher volunteers as one principal (Participant 10) explained: “We do not have our own after school program, so anything we do it’s completely volunteer, teachers volunteering.” The principal (Participant 10) went on to say: “It would be really cool if we had more soccer, more volleyball, more flag-football…because I know they (students) would do it in a heartbeat, they (students) would love to do it. We just do not have the funding for it.” Schools also struggled to maintain after school programs because of funding challenges as one teacher (Participant 11) explained: Last year we lost our 21st century grant that gave us an after school program. We had soccer. We had tennis. We had running. We had all kinds of things last year when we had the grant. But since we lost the grant, we do not have a lot of after school as much as we did before.”

### Quantitative results

A total of 346 people responded to the survey (33.6% response rate). The final analytic sample consisted of 247 respondents (59 people were ineligible, two people completed the survey twice, and 38 opened the survey but did not complete it). Participants were from 22 elementary schools (mean respondent/school = 11, range = 1–20) as three principals opted their school out of the study, and one principal completed the survey but opted their staff out of the study. Of the participating schools, 59.0% (*N* = 13) were Title I meaning ≥40% of their students were economically disadvantaged. Characteristics of respondents are presented in Table 3.

**Table 3:** Individual-level respondent characteristics.

Variable	Total Sample (*n* = 247)
*Gender (%, n)* ^a^	
Female	95.4 (186)
Male	4.6 (9)
Age (m, SD)^b^	40.1 (11.5)
Years in current job (m, SD)	6.6 (6.8)
Years working in education (m, SD)	13.5 (9.5)
*Job type (%, n)*	
Classroom teacher	68.8 (170)
Physical education teacher	9.7 (24)
Administrator (principal/assistant principal)	2.8 (7)
Support staff	18.6 (46)

a 195 participants answered gender question.

b 193 participants answered age question.

**Figure 2:**
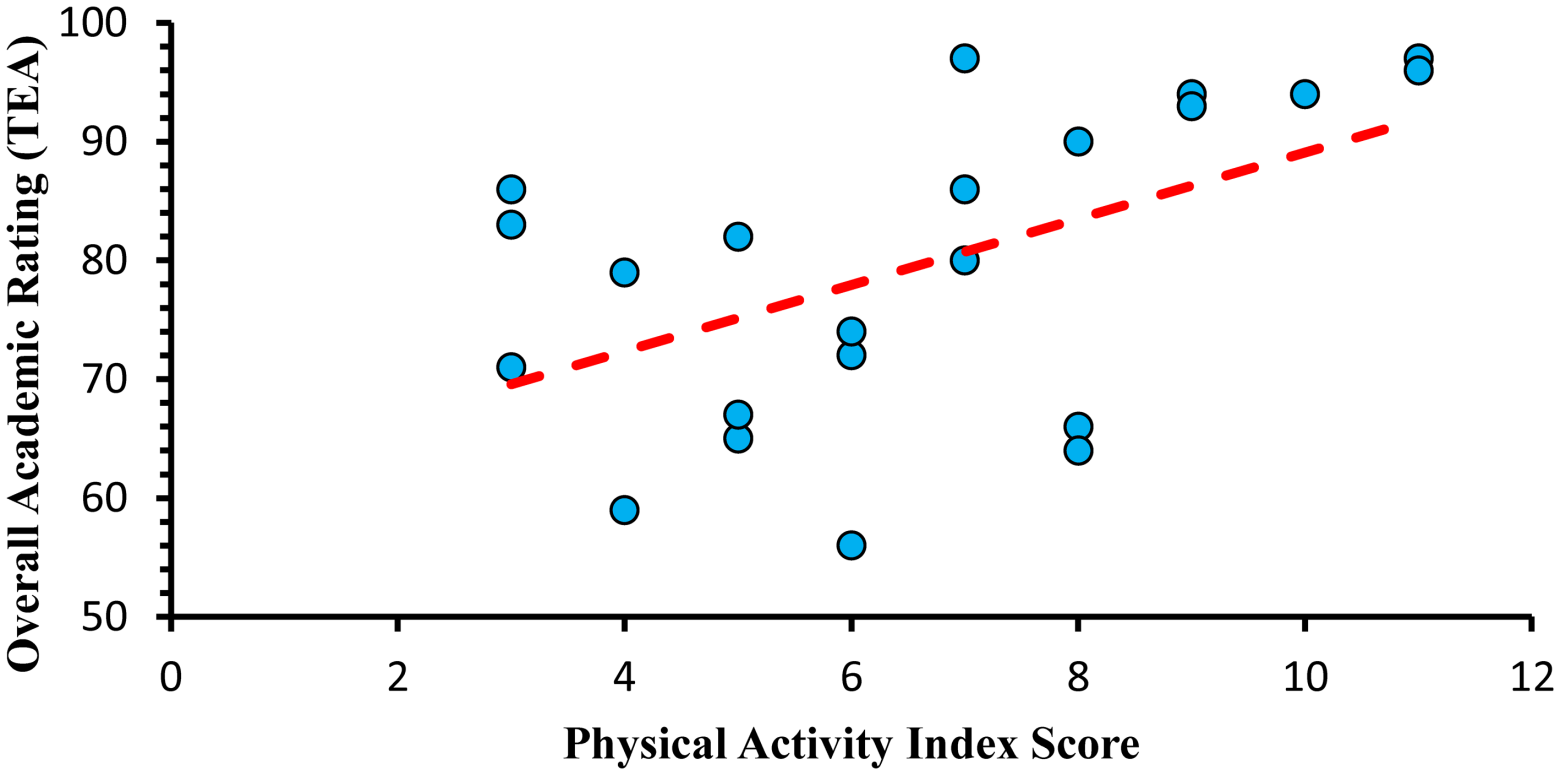
Association between school academic ratings and physical activity index scores.

Table 4 provides school-level implementation trends across whole-of-school components, each school’s calculated PA rating, and TEA accountability rating (A–F). Schools offered 2–3 days of PE/week, and an average of 135 min/week across the district. Six schools used an alternating PE schedule meaning students had two days of PE one week and three another week. All schools offered daily recess with an average of 135 min/week across the district. Classroom-based approaches were inconsistently used, with brain breaks/active learning more commonly used than motorlabs. On average, schools were providing accessible after school programs to their students. Although fewer schools reported accessible before school programs (compared to after school) and inconsistently promoted active transport. Figure 2 illustrates there was a moderate, direct association between the school-level PA index score and the TEA accountability rating, *r*(22) = 0.53, *p* = 0.01.

**Table 4:** Quantitative results table—school-level implementation.

School (*n*)	PE mins/week(mode)	Recess min/week (mode)	Classroom-based approaches		After-school *M* (SD)	Before school *M* (SD)	Active transport M (SD)	PA index score	TEA grade
			Brain breaks /active lessons *M* (SD)	Motorlab *M* (SD)					
School A (13)	100	100	2.5 (0.9)	1.3 (0.5)	3.9 (1.3)	2 (1.6)	3 (0.8)	3	B
School B (13)	135	75	2.7 (0.9)	1.5 (0.5)	3.4 (0.9)	2.2 (1.3)	2.8 (1.1)	3	B
School C (9)	112.5^a^	150	2.6 (1)	2.8 (1.2)	3.3 (1.5)	1.8 (1.4)	2.6 (0.9)	3	C
School D (12)	100	100	3.1 (1.4)	3.3 (1.2)	3.4 (1.4)	1.8 (1)	2.1 (0.9)	4	C
School E (10)	137.5^a^	75	3.5 (1.1)	2.3 (1)	3.2 (1.5)	2.4 (1.2)	3.1 (1.2)	4	F
School F (17)	90	150	2.3 (1)	1.7 (0.8)	4.3 (0.9)	2.8 (1.5)	3.2 (0.8)	5	B
School G (9)	112.5^a^	150	2.4 (0.9)	1.3 (0.8)	4.6 (0.5)	2.1 (1.6)	3.3 (1.2)	5	D
School H (14)	135	75	2.9 (1.2)	2.5 (1.1)	4.8 (0.4)	2.8 (1.6)	3.2 (1.2)	5	D
School I (2)	137.5^a^	75	2.5 (0.7)	2.5 (0.7)	4 (0)	4 (0)	3.8 (0.4)	6	C
School J (11)	135	150	2.7 (1)	2.1 (1.1)	4.1 (1.2)	1.9 (1.5)	2.4 (1.2)	6	F
School K (6)	137.5^a^	150	2 (0)	2.2 (0.5)	4.3 (0.8)	3 (1.1)	2.2 (0.7)	6	C
School L (4)	165	150	2.5 (1.3)	1.3 (0.5)	4.3 (1)	3.3 (1.7)	2.8 (1.3)	7	B
School M (13)	125^a^	150	2.8 (0.9)	2 (0.4)	4.8 (0.4)	3.9 (1.3)	4.5 (0.8)	7	A
School N (11)	125	150	3.4 (1.1)	2.6 (1.1)	3.1 (1.3)	4.8 (0.4)	3.9 (0.9)	7	B
School O (5)	180	150	2.4 (0.6)	1.2 (0.5)	4.4 (0.6)	4.8 (0.5)	2.0 (0.7)	8	D
School P (16)	150	150	3.1 (1)	1.6 (1.2)	4.1 (1.2)	2 (1.2)	3.8 (1.1)	8	A
School Q (11)	135	150	3.2 (1.2)	2.9 (1.3)	4.1 (1.2)	1.8 (0.9)	3.1 (1)	8	D
School R (20)	165	150	3.5 (1.2)	1.8 (1.5)	4.4 (0.5)	1.9 (1.5)	4.1 (1.1)	9	A
School S (13)	135	150	3.3 (1.1)	1.9 (1.1)	4 (1.6)	2.2 (1.7)	4.1 (1)	9	A
School T (1)	165	225	5	2	4	Missing	4.5	10	A
School U (20)	150	150	3.4 (1.1)	1.8 (0.6)	4.4 (1)	4.7 (0.8)	4.8 (0.5)	11	A
School V (17)	150	150	3.6 (0.7)	1.3 (0.6)	4.5 (1)	4.5 (1.2)	4.3 (0.7)	11	A
Mean (SD)	135 (23)	135 (37)	3.0 (0.6)	2.0 (0.6)	4.1 (0.5)	2.9 (1.1)	3.3 (0.8)	6.6	3.4
Range	90–180	75–225	2.0–5	1.2–3.3	3.1–4.8	1.8–4.8	2.0–4.8	3–11	1–5

a Schools used an alternating PE schedule meaning the weekly minutes are averaged over 2 weeks.

### Joint display results

**Figure 3:**
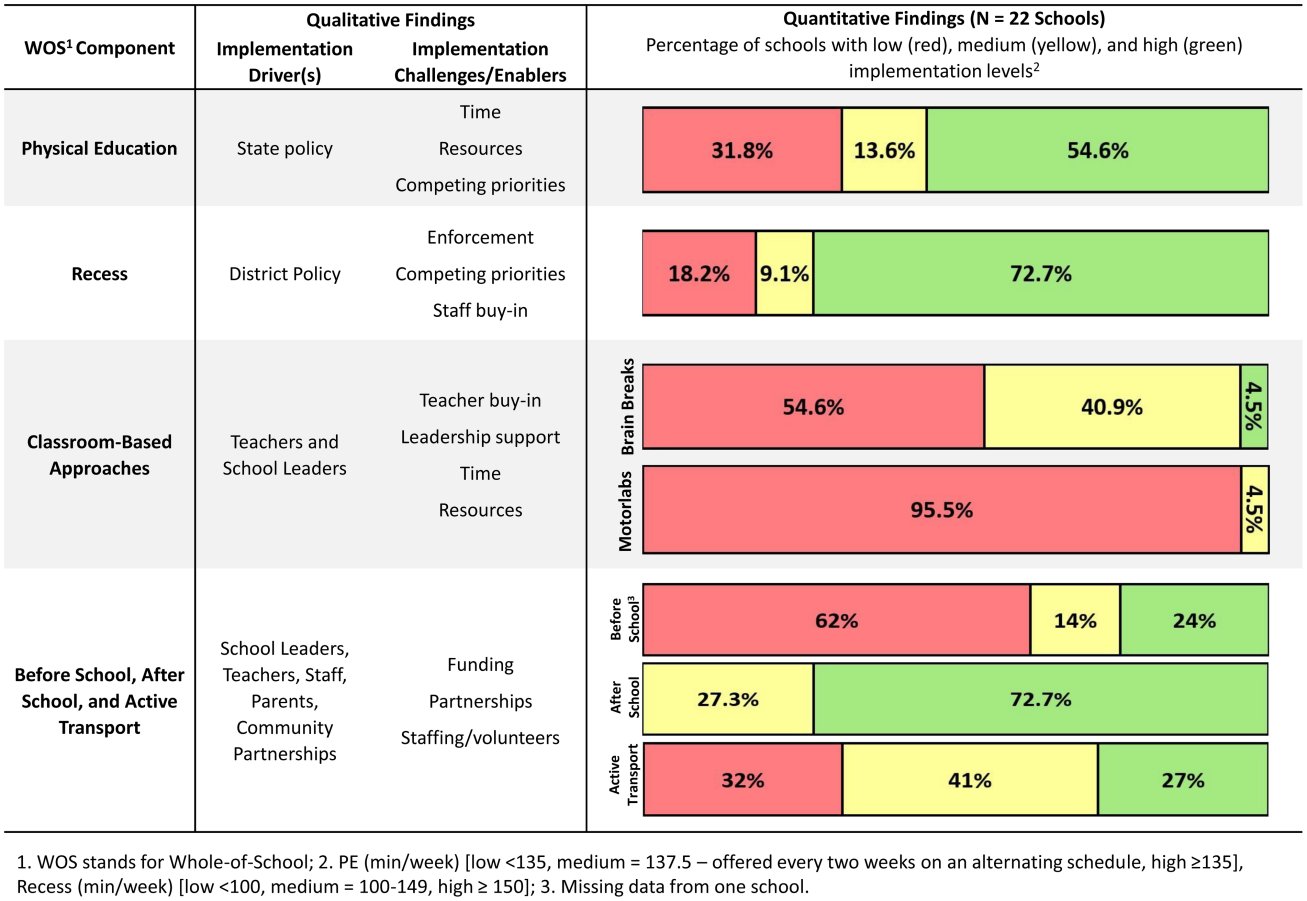
Joint display of whole-of-school implementation.

Figure 3 provides a joint display with district-level implementation trends and key qualitative findings. Notably, 54.6% of schools were in full compliance with the state’s PA policy for PE and 72.7% complied with the district’s recess policy. These are consistent with qualitative findings suggesting some schools were out of compliance. Additionally, qualitative findings indicated there were implementation challenges with classroom-based approaches. Even though schools started motorlabs, they were not widely used by teachers. The quantitative findings indicated 45.4% of schools reported medium/high use of brain breaks or physically active learning, and 4.5% of schools reported medium/high use of motorlabs.

Qualitative data indicated before school programs were not always available at schools. Consistent with qualitative findings, 23.8% of schools reported having high accessibility to before school programs. All schools reported having medium/high accessibility to after school programs and 68.2% of schools reported medium/high levels of promoting active transport. These results were inconsistent with qualitative findings, which indicated schools struggled to maintain after school programs, and there was little discussion about active transport promotion.

## Discussion

This study used a mixed methods design to examine the implementation of PA approaches in elementary schools. Schools promoted PA in a manner consistent with a whole-of-school approach, although there was wide variation. Schools also leveraged existing infrastructure (e.g., partnerships with after school providers) and accessible resources (e.g., GoNoodle, CATCH, SPARK) to support their efforts. PE and Recess were implemented top-down as their scheduling was driven by state and district policies. In contrast, classroom-based approaches were primarily implemented bottom-up by teachers and school leaders. Additionally, schools drove the use of before and after school approaches, and the promotion of active transport. Notably, there was a direct association between the school PA index score and TEA academic accountability ratings, suggesting that top performing academic schools were often providing the most PA opportunities throughout the day.

Previous research examining the use of PA approaches among a nationally representative sample found that a majority of schools offered at least 20 min of daily recess; yet daily PE (or ≥150 min/week), before and after-school programs, and classroom activity breaks were less commonly implemented (19). School-based PA approaches are challenging to implement due to competing priorities, limited staff capacity, a lack of resources/support, and unsupportive cultures (11, 20, 21). Subsequently, implementing a whole-of-school approach (or CSPAP), where many components are employed together, can be especially difficult.

Our study highlights many of the previously reported implementation barriers even though this was not a primary objective nor a comprehensive list. Our study also illustrates how each whole-of-school component can present its own challenges and may require tailored solutions. For example, common barriers to more top-down approaches like PE include competing priorities and a lack of resources (22). Thus, working with school and district leaders to restructure schedules and the allocation of resources may be optimal in these instances. In contrast, bottom-up approaches like classroom-based approaches benefit from teacher buy-in, and common implementation barriers include a lack of teacher knowledge, self-efficacy, and/or motivation (23). Thus, providing teacher trainings and leadership support may be optimal when promoting classroom-based approaches (24).

Our findings illustrate that a supportive culture plays an important role in the successful implementation of both PE and classroom-based approaches. Creating a supportive culture may begin with ensuring school leaders, teachers, and staff understand and value the connections between PA, student behavior, and learning. Establishing these connections can help align health and academic priorities rather than having them compete. In our study, six of seven A-rated schools offered the most physical activity opportunities throughout the school day. These findings illustrate that schools can maintain strong academics while providing multiple opportunities. Alternatively, the A-rated schools may also have had less academic pressure, and thus were more comfortable providing various PA opportunities. More work is necessary to understand this connection and whether providing more PA opportunities improves school academic ratings.

The longstanding challenge of building an activity-centered culture to implement sustainable PA approaches in schools has initiated a paradigm shift in how to develop these programs. One example is the Creating Active Schools Framework (CAS) (25). The CAS used a co-design approach in which researchers work with teachers and school leaders, to develop a framework centered on a “whole-school” practice and ethos. This ethos drives policy and vision for promoting PA in schools. The CAS also highlights the need to address different levels of school systems (i.e., district, schools, and individuals) through a connected, systems-thinking approach rather than focusing on the development and “push” of single-element interventions. Our findings align with the paradigm shift proposed by the CAS Framework, by highlighting that schools are promoting PA through multiple approaches, and increasing one approach may come at the expense of another. Further, we found the top-down and bottom-up approaches may require engagement with different actors within the school system. Thus, leveraging existing assets while providing tailored implementation strategies that account for the entire school ecosystem may be most beneficial to schools.

Other studies have focused on providing implementation support for PA approaches in schools (26–29). For example, Be a Champion! (BAC), leverages implementation frameworks and strategies to guide CSPAP implementation in schools (20). Despite mixed results, the approach uses key strategies such as identifying a champion, conducting a needs/resource assessment, building capacity, and developing an implementation plan (20, 30). This work highlighted the differences between participating schools, and the importance of understanding barriers, strengths, resources, and perceived benefits. These lessons align with our findings, which indicated that schools responded to their unique context with different configurations in the number and types of PA opportunities offered. Overall, more work is necessary to understand how to best support implementation of whole-of-school components.

### Limitations

We collected study data prior to the COVID-19 pandemic. Schools may have since changed how they support PA given the pressure to make up for learning losses during the pandemic. Additionally, we used participant recommendations to recruit the qualitative sample, which may have led to biased perspectives of PA and implementation. The survey data were from a convenience sample and schools had a wide range of respondents (1–20), which may impact the validity and reliability of results. Specifically, the values from schools with fewer respondents may be less reliable and valid because they are sensitive to a single or small group of representatives within a school. The quantitative survey was also self-report, which opens the potential for social desirability/recall bias. We also found variation among survey responses within schools (e.g., some participants reported 2-whereas others reported 3 days of PE). The variation could be an indicator that not all students (or grades) had the same amount of recess or PE within a school, or that there were recall issues. Further, the measures were developed through the qualitative work but lack psychometric testing. Lastly, data are from a single school district in Texas, which limits generalizability.

### Strengths

The mixed method design allowed for a richer understanding of what and how PA approaches were implemented, while examining district-level trends. We leveraged data from multiple sources to examine the association between school-level PA support and academics. We worked with district-level partners from project inception to completion. Our district partners helped guide project goals, develop the interview guide and survey, recruit participants, and provide feedback about preliminary findings. Establishing meaningful partnerships meant to bridge the research to practice gap is critical for advancing PA promotion in schools. Our study further illustrates the early stages of partnership building by examining PA approaches used in practice, how they are implemented, and how they relate to school-level academics. Our study also included perspectives from multiple positions within schools (administrators, teachers, and staff), which helped highlight the various roles school members play when implementing PA approaches. Further, our study focused on understanding implementation approaches that schools were using in practice, and not as part of an intervention study. Thus, our findings provide valuable insights about “real world” implementation of PA approaches in schools.

## Conclusion

Schools are uniquely positioned to support student’s PA, health, and learning. Our study showcases how schools promote PA in practice, consistent with a whole-of-school approach. Yet many schools struggled with high levels of implementation due to common challenges. Based on our findings, there is a need to build meaningful partnerships with schools to enhance their implementation of ongoing PA approaches. Future work should also continue to examine how school-based PA opportunities relate to schools’ academic performance given academics are a key factor influencing implementation. Our study also highlights how schools can benefit from a holistic approach that meets a school’s specific needs, leverages their assets, accounts for dynamic environments, and allows school partners to drive solutions.

## Data availability statement

The original contributions presented in the study are included in the article/Supplementary materials, further inquiries can be directed to the corresponding author.

## Ethics statement

The studies involving humans were approved by The Committee for the Protection of Human Subjects at the University of Texas Health Science Center at Houston approved this study (HSC-SPH-17-0980). The studies were conducted in accordance with the local legislation and institutional requirements. The participants provided their written informed consent to participate in this study.

## Author contributions

TW led the overall conception and design of the study. TW and MR collected and analyzed the data. TW drafted the manuscript with support from CP and DC. NH contributed to the conception of the qualitative analytic approach. CP, DC, and JB helped with data interpretation. All authors contributed to the article and approved the submitted version.

## Funding

During the writing of this manuscript, Dr. Walker was supported by the National Heart, Lung, and Blood Institute (1K01HL151817-01A1). Data collection was supported by a research career development award for (K12HD052023): Building Interdisciplinary Research Career in Women’s Health Program-BIRCWH; Berenson, PI) from the Eunice Kennedy Shriver National Institute of Child Health and Human Development (NICHD) at the National Institutes of Health and the University of Texas Health Science Center at Houston School of Public Health Cancer Education and Career Development Program grant from the National Cancer Institute (R25 CA057712). During the writing of this manuscript Dr. Craig was supported by the University of Texas Health Science Center at Houston School of Public Health Cancer Education and Career Development Program grant from the National Cancer Institute (T32/CA057712). This work is partially supported by the Center for Health Promotion and Prevention Research.

## Conflict of interest

The authors declare that the research was conducted in the absence of any commercial or financial relationships that could be construed as a potential conflict of interest.

## Publisher’s note

All claims expressed in this article are solely those of the authors and do not necessarily represent those of their affiliated organizations, or those of the publisher, the editors and the reviewers. Any product that may be evaluated in this article, or claim that may be made by its manufacturer, is not guaranteed or endorsed by the publisher.

## Disclaimer

The content is solely the responsibility of the authors and does not necessarily represent the official views of the funding agency.
