# Supplementary material for: Elementary school staff perspectives on the implementation of physical activity approaches in practice: an exploratory sequential mixed methods study
Source: Front Public Health. 2023 Aug 24;11:1193442. doi: 10.3389/fpubh.2023.1193442 (PMC10483115; doi:10.3389/fpubh.2023.1193442)
Supplement: Supplementary file 1 [file Table_1.DOCX]

In Depth Interview Guide

Warm-up Questions

1. What is your current position?
2. How long have you been working at your school?
3. What do you like most about your position?

Programming-related questions

1. How do you feel about the amount of physical activity students are getting at your school?
2. Tell me about the current physical activity policies at your school
   1. Probe: how well are policies adhered to?
3. Tell me about the current physical activity approaches at your school
   1. Probe: How effective are the approaches

Implementation

1. How are physical activity approaches selected to be used?
   1. Probe: who is involved in the decision making?
   2. Probe: what resources are available to support decision making?
2. How do you feel about the process for delivering programs?
   1. Probe: who is involved in delivering programs
   2. Probe: what resources are available?
   3. Probe: what strategies help delivery?
3. What makes it difficult to deliver physical activity programs?
4. What makes it easier to deliver physical activity programs?
5. What makes an approach stick?
